# Fake review identification and utility evaluation model using machine learning

**DOI:** 10.3389/frai.2022.1064371

**Published:** 2023-01-19

**Authors:** Wonil Choi, Kyungmin Nam, Minwoo Park, Seoyi Yang, Sangyoon Hwang, Hayoung Oh

**Affiliations:** ^1^Department of Business Administration, Sungkyunkwan University, Seoul, South Korea; ^2^College of Computing and Informatics, Sungkyunkwan University, Seoul, South Korea

**Keywords:** machine learning, fake review, fake review detection technique, e-commerce, useful reviews, SVC, logistic regression

## Abstract

Due to the structural growth of e-commerce platforms, the frequency of exchange of opinions and the number of online reviews of platform participants related to products are increasing. However, given the growth of fake reviews, the corresponding growth in the quality of online reviews seems to be slow, at best. The number of cases of harm to retailers and customers caused by malicious false reviews is steadily increasing every year. In this context, it is becoming difficult for users to determine useful reviews amid a flood of information. As a result, the intrinsic value of online reviews that reduce uncertainty in pre-purchase decisions is blurred, and e-commerce platforms are on the verge of losing credibility and traffic. Through this study, we intend to present solutions related to review filtering and classification by constructing a model for judging the authenticity and usefulness of online reviews using machine learning.

## 1. Introduction

Thanks to the development of IT technology, the global e-commerce market is achieving structural growth (CAGR +17.78%) (Statista, 2022). Traffic within e-commerce platforms is exploding, and online shopping transactions are also increasing every year. In addition, due to the nature of online shopping malls, users' need to obtain useful product information through online reviews have increased, and the number of online reviews has naturally increased as online retailers provide review platforms that enable users to interact freely.

The increase in the number of reviews soon led to a strengthening of the influence of reviews on purchasing decisions (Jung, 2020). Users can now more easily obtain comprehensive information about a product through extensive reviews. As satisfying purchase experiences of individual users based on product reviews have increased, the customer dependence on reviews at the time of purchase has also increased. According to related statistics, 93% of users are directly or indirectly affected by online reviews in the purchasing decision-making process (Invesp, 2022). As such, reviews have become a key factor in determining whether users buy or not, and at the same time, they have a direct impact on the sales and reputations of vendors (Barbado et al., 2019).

However, the increase in the number of reviews inevitably leads to difficulties in controlling and monitoring reviews, which increases macroscopic false reviews within a platform. Deceptive spam reviews that abuse the importance of reviews to manipulate product reputations are increasing, blurring the purpose of a review to provide useful information for purchasing a product (Cha et al., 2021). According to an article, part-time job openings for creating false reviews in the industry are found to be widespread (Jung, 2020), and the increase in false reviews actually written is also confirmed to be steep. According to the self-data of Baedal Minjok, a delivery application service, false reviews exceeded 100,000 as of 2020 last year from 20,000 in 2019 (Cha et al., 2021).

**Figure 1:**
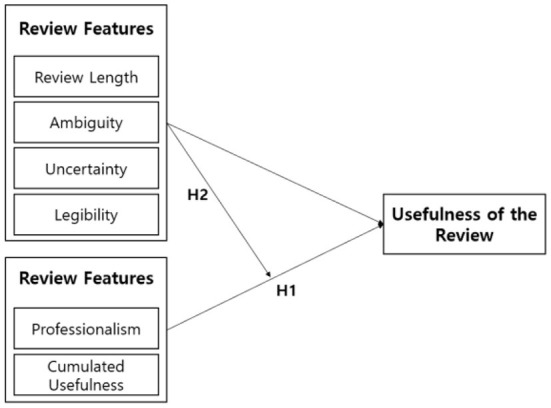
Analyzing usefulness of a review.

False reviews adversely affect both consumers and vendors, and consequently reduce the reliability of the platform itself. According to a survey by Invesp, 54% of consumers do not purchase products if they find false reviews, which leads to a direct decline in sales by vendors (Invesp, 2022). In this increasingly common scenario, participants broadly lose trust in the entire platform, resulting in platform departure (Chengzhang et al., 2014). Figure 1 shows how we specifically analyzed the usefulness of the reviews on a product.

Therefore, there is a need for various solution proposals that can block this vicious cycle. This study proposes an algorithm that utilizes machine learning to (1) remove macroscopic views of fake reviews and (2) place reviews that provide useful information to buyers at the top of displayed platform results. In this study, various machine learning algorithms for classifying good and fake reviews are presented and their accuracies are compared. It is expected that the best proposed solutions can provide benefits to all platform participants.

## 2. Prior research

### 2.1. Natural language processing technology

Natural Language Processing (NLP) is a field in which computers analyze and process the meaning of natural languages and study the interactions between humans and computer languages. Key areas of deep learning-based natural language processing include language models, text classification, text generation, document summary, question answering, and machine translation. The key deep language processing techniques are Neural Networks (NNs), Recurrent NNs, Network-Analect (NNN). Transformers, Generative Pre-Trained Transformers (GPT), and Bidirectional Encoder Represents (BERT) models are also used in NLP research (Table 1).

**Table 1:** Prior studies used in this study.

	**Title**	**Published in**	**Subject**	**Characteristics**	**Dataset**	**Limitations**	**Implications**	**Used Features**
**1**	Analysis of the Status of Natural Language Processing Technology Based of Deep Learning	The Korea Journal of BigData, vol 6 no 1, 2021, pp.63–81	AI and classification of NLP technologies	Focuses on an artificial intelligence (AI) classification system and discusses the status of patent-based technology development	X	This is a review article that organizes theoretical content and does not provide new technology or research	Summary of artificial intelligence and natural language processing technologies developed and advanced thus far	Considers using BERT and RNNs among natural language processing techniques
**2**	How to improve the accuracy of recommendation systems: Combining ratings and review texts sentiment scores	Journal of Intelligence and Information Systems	Text sentiment analysis using a sentiment dictionary	Quantifies reviews, which are qualitative data, to improve the accuracy of judgment algorithms	Crawling user ratings and review data provided by “Naver Movie”	As an algorithm specialized in the movie domain, it lacks versatility, and there are technical limitations that make it difficult to apply adverbs in advance.	Demonstrates the possibility of using a quantitative data analysis methodology for qualitative data analysis	Proposed a new rating Labeled as 0 (negative) or 1 (positive) based on the scoring criteria
**3**	Product Review Data and Sentiment Analytical Processing Modeling	The Journal of Society for e-Business Studies, vol 16 no. 4, (2011), pp.125–137	Suggests a way to dynamically analyze and integrate opinion information online through a sentiment analysis processing modeling	Suggests a way to utilize OLSAP while complementing the shortcomings of existing sentiment analyses.	Crawling application evaluation data of LG U+'s OZ store	Compared to various experiments, the advantages and characteristics of the new modeling method are not properly demonstrated.	It is expected that it can be used for various purposes, not only in product evaluation, but also in combination with e-commerce systems in the future.	Diversification of opinion data expression methods
**4**	A Study on the Impact of Consumer Review on Mobile Commerce Performance	Global Business Administration Review, vol 17, no 6	Analysis of how quantitative and non-quantitative characteristics of mobile commerce customer reviews affect sales performance and preference	Understanding how review characteristics affect preferences and purchasing decisions through crawling, LDA, and sentiment analysis	Review data of 558 men's t-shirts for sale on Shopee	It is difficult to generalize, as only the Singapore platform and men's t-shirts were analyzed as examples.	In commerce, it was confirmed that non-quantitative customer reviews, emotionally analyzed through quantitative and textual information about products, can affect performance.	LDA analysis, sentiment analysis to measure non-quantitative characteristics, linear regression analysis
**5**	A study on the Detection of fake reviews using Machine Learning	2020 Dissertation at Chonnam National University	Presenting a machine learning model to judge fake reviews according to variables	Unlike the existing text-oriented analyses, predictability is confirmed by using the structural characteristics and behavior of blog posts for analysis.	500 samples of review data presented in Naver blog posts according to 11 variables	Data is limited to blogs, so it is difficult to apply it to various SNS Low accuracy, as the review data is limited to 500 samples.	Efficient and effective prediction is possible in terms of speed and accuracy, and the possibility of using machine learning in similar research fields that do not have objective discrimination criteria has been confirmed.	Conducts ROC analysis to evaluate effectiveness and checks AUC values to verify accuracy, determines features important in screening fake reviews
**6**	A Study on the Effect of Reviewer's Attributes on the Usefulness of Online Reviews	Korean Association of Information systems, The Journal of Information systems, vol 29, no 2, (2020), pp.173–195	Analysis of review characteristics that enhance the usefulness of the review	Interaction and moderating effects between review attributes and reviewer attributes are derived through regression and empirical analysis	3,000 online reviews of restaurants	Since the variables established in the above study do not represent the characteristics of the review, it is necessary to establish a universal hypothesis.	By analyzing the usefulness of reviews that can be somewhat abstract in a scientific way, the possibility of constructing a mechanical model is presented.	The mutual relationship between each variable is divided into a positive (+) relationship and a negative (–) relationship.

(Chengzhang et al., 2014; Lee et al., 2014; Yang and Fan, 2014; Barbado et al., 2019; Jung, 2020; Cha et al., 2021; Lee and Park, 2021).

BERT learns by masking words at random locations in a sentence based on a two-way Transformer encoder, using an attention model to determine the context of a word in the entire sentence to predict the missing word(s). GPT is also a transformer-based model, but GPT masks the right words in the form of predicting the next word using only the preceding word, and BERT masks any word in the sentence to use the attention from any word that constitutes the sentence. In this study, BERT wasn't used to determine the authenticity of the review, because the Korean language processing package (KoNLPy) was thought to be more suitable and efficient for this study in its handling of Korean natural language processing (Park, 2021).

### 2.2. Dictionary-based text analysis

The dictionary-based analysis method is a methodology that quantifies reviews by matching review data that has undergone a preprocessing process to a pre-built emotional dictionary. Dictionary-based analysis methodologies are actively used to analyze qualitative data, such as user review text. Therefore, we investigated (Hyun et al., 2019), a related prior paper.

This study (Hyun et al., 2019) improved the accuracy limitations of existing recommendation systems by creating an algorithm that combines the user's emotions contained in the review data with the rating, which is a quantitative value. In particular, the quantitative data were more precisely corrected by scoring the emotional values calculated through emotional analysis using a dictionary. Emotional figures calculated through the Emotional Dictionary are reflected in the ratings to derive a new proposed new rating, which is utilized in the system to enable more sophisticated recommendations and predictions.

Hyun et al. (2019) is meaningful in that it provides a method of processing qualitative data into quantitative data. This methodology that is optimized for quantitative data analysis can be applied to this study. In particular, Hyun et al. (2019) also suggested the possibility that K-Nearest Neighbors (KNN) can be applied to algorithms to improve the accuracy of the model. However, since the emotional dictionary built by Hyun et al. (2019) is a dictionary specialized in the movie domain, there is a limitation in terms of versatility, so improvement is needed. In addition, the technical limitation of the study is that adverbs, which are important parts of language that represent the intensity of the meaning of emotional expression words, were not reflected in advance.

### 2.3. Sentiment analysis processing modeling

Opinion data is extracted from text data that mentions the user's opinion about the object itself or some features of the object in a sentence, such as “This software updates really fast.” In the case of Korean, this method uses “morphology analysis” and “phrase analysis” as a pre-processing step. Opinion text extracts information, such as the opinion expression, object, and modifier, after proper natural language processing, such as morpheme analysis and parsing, and then determines whether the meaning of a specific word is positive/negative, and strengthens or weakens the meaning to obtain a final polarity value. OLSAP sees the polarity information of the opinion data as an element corresponding to a “measurement”, such as sales volume, uses polarity information in the opinion data, and obtains complex information, such as users' overall evaluation of products, regional evaluations, and changes in opinions over time (Myung et al., 2008).

### 2.4. An analysis of e-commerce review using emotional analysis and linear regression analysis

In the study, data related to sales, preference, price, and reviews of 1,000 men's yoga T-shirts sold at Shopee, established in Singapore, were crawled (Jung et al., 2020). In order to extract the non-quantitative characteristics of the review, the product description and the corpus of consumer reviews were analyzed frequently and classified into four characteristics such as overlapping size, price, material, and delivery among the top 20 frequencies of the two groups. LDA analysis was conducted on the subject of four predefined quality characteristics, and keywords for each characteristic were extracted and emotionally analyzed to measure the non-quantitative characteristics of the product. Finally, through linear regression analysis of the preference and sales performance, it was possible to derive the research results that the average score among the quantitative review characteristics had a significant effect on sales performance.

This study is significant in that it conducted an empirical study that concluded that the quantitative and non-quantitative characteristics of customer reviews referred to by customers for decision-making in the recent growing mobile commerce field affect sales performance (Jung et al., 2020). This confirms that e-commerce can affect performance through quantitative and textual information regarding products, and suggests the need to recognize the importance of customer text reviews in future platform performance and analyze their impact on performance.

### 2.5. Quantitative analysis of blogs using a random forest

In this study, various variables related to the objective variable were selected and used as learning data to use traces of habitual patterns formed by continuous behavior as quantified measurement tools to detect fake reviews (Lee, 2020). In addition, in order to determine whether or not an advertising review was conducted, a judgment was made through the detection of words repeatedly used in advertising reviews. The research was conducted as follows. After accessing the post using Python to collect data on the Naver blog, web crawling was performed by adding Tesseract-OCR to digitize some data and extract characters in images to store them as data. Data preprocessing was performed through a total of four stages. The program was produced and proceeded to automatically categorize and digitize the extracted values in the data collection process, and missing values, outliers, and duplicate data were removed. Finally, 500 datasets were extracted through downsampling. As a result of the analysis, it was confirmed that the area under the Receiver Operating Characteristic (ROC) of the random forest converges the most at 1, and the Area Under Curve (AUC) value is also 0.913, which makes the most accurate prediction.

**Figure 2:**
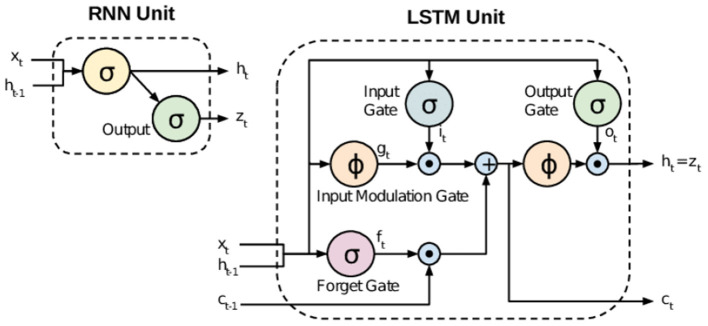
Comparison between RNN and LSTM.

It is meaningful that more efficient and effective prediction of a false review, in terms of speed and accuracy, is possible through the research in Lee (2020) than the methods used in previous studies. Figure 2 shows how we proceeded with the study based on the ideas of the prior researches.

### 2.6. Analyzing review usefulness

Reviews have the purpose of providing useful information to buyers and sellers. First, this research team aimed to judge whether their research model could determine the falsehood of a specific review. The second aim was to judge whether a review provides useful information for platform participants' decisions, as high-quality reviews help all participants. Therefore, we investigated the related previous studies that analyzed the usefulness of reviews (Lee and Park, 2021).

Yao et al. (2020) sets the variables that determine the usefulness of a review, as shown in [Chart A]. After that, in order to verify the research model, the research hypothesis was established by appropriately combining variables, and the research hypothesis was verified by Tobit regression analysis and empirical analysis.

Through the study, (1) the relationship between review attributes and review usefulness, and (2) the moderating effect of review attribute variables were derived. This research team also strengthened the effectiveness of the model by establishing the team's own criteria for judging the usefulness of reviews based on the previous research results of Yao et al. (2020).

## 3. Used algorithms

### 3.1. LSTM

Developed in 1986, the Recurrent Neural Network (RNN) provides a technique for analyzing the sequential dynamic characteristics of cyclically connected units as a type of neural network. RNNs have less long-term predictive capability as they only work on relatively short sequences due to the long-term dependency problem. Fortunately, Long Short-Term Memory (LSTM) NNs improve this shortcoming of the RNN through the addition of cell state values.

**Figure 3:**
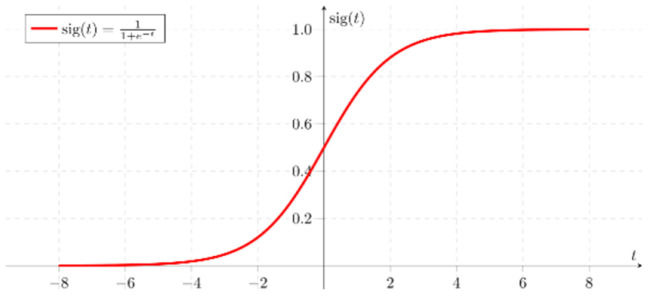
Sigmoid function used in logistic regression.

In the case of the RNN, only the information from the previous time step is conveyed to the next cell, indicating only short-term memory, but in the case of LSTM, both long and short-term memories are present and displayed in the conceptual diagram with two arrows. The LSTM has a more sophisticated structure than RNNs by delivering information divided into long and short periods. Figure 3 shows overall architecture of LSTM model.

### 3.2. Agglomerative clustering

Agglomerative clustering is an algorithm that clusters in a bottom–up manner while each subset of data is divided, starting with small units, and it then combines all data together until a specified number of clusters remain. The methods of combining the two clusters include Ward, Average, and Complete.

### 3.3. SVC

The Support Vector Machine (SVM) is a supervised learning model that is mainly used for classification and regression analysis. The SVM uses support vectors to define decision boundaries and to classify unclassified points against those decision boundaries.

SVC refers to the SVM model used for Classification, which is characterized by the fact that it works well if the data are linear, but may not if it is nonlinear.

### 3.4. KNN

The K-Nearest Neighbor (K-NN) algorithm is a distance-based classification analysis model that measures the distance between new data and existing data and determines new data types. Data with similar characteristics are used under the assumption that they tend to belong to similar store owners, and the K-NN has the advantage of being efficient in image processing, letter recognition, face recognition, and product recommendation algorithms.

### 3.5. Logistic regression

“Logistic Regression” is a type of regression technique that can be performed if the dependent variable is dichotomous in a predictive analysis and is used to quantify the relationship between one or more dependent binary variables and one or more numeric, nominal, and ordinal independent variables (Lee et al., 2014).

In the logistic regression formula, the relationship between the dependent variable and one or more independent variables is expressed in the following Equations (1) and (2).


(1) $$\begin{array}{l} z=b_{0}+\;b_{1}x_{1}+\;b_{2}x_{2}+\ldots +b_{n}x_{n} \end{array}$$

(2) $$\begin{array}{l} P=\;\frac{1}{\left(1+e^{-z}\right)} \end{array}$$

In Equation (1), *b*_0_ is the intercept of the model, and *b*_*i*_ (*i* = 0, 1, 2, …, *n*) is the slope coefficient of the logistic regression model.

**Figure 4:**
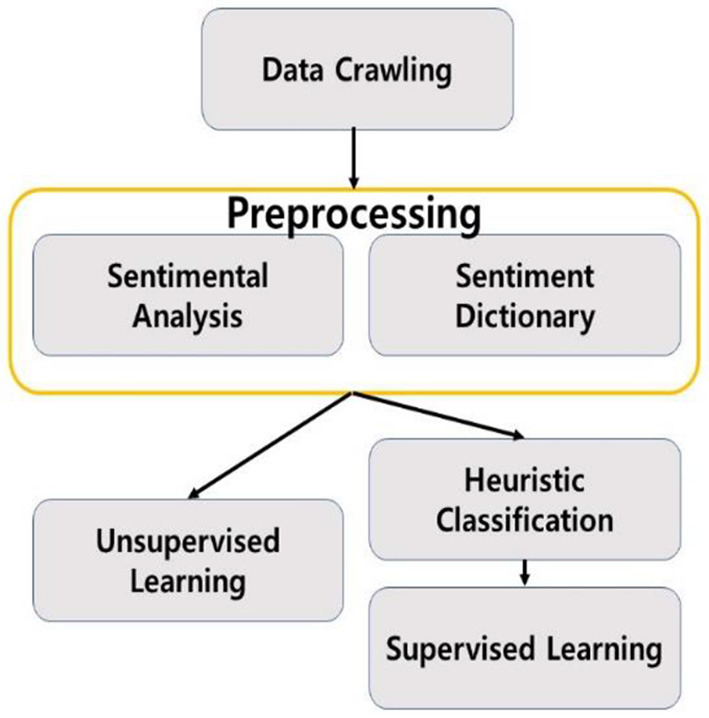
Flowchart of the study.

This shows that *x*_*i*_ (*i* = 1, 2, …, *n*) is an independent variable. In addition, in Equation (2), P is the probability that the event will occur and e is Euler's number. By applying these several independent variables (xi) to Equations (1) and (2), which are logistic regression formulas, the probability (*P*) of an event occurring can be determined (Statista, 2022). Figure 4 shows sigmoid function used in Logistic Regression.

### 3.6. LGBM

The Light Gradient Boosting Model (LGBM) is a tree-based learning algorithm model using the Gradient Boosting (GB) technique, which combines classifiers vertically, creating and combining trees through the Gradient Boosting method, and then predicting targets. The Leaf-Wise method has the advantage of short learning time, low memory use, and fast hyperparameter tuning.

## 4. Methods

The study was conducted in the following figure of a flowchart.

### 4.1. Data description

All the data necessary for this study was obtained through crawling using “selenium” on the Naver shopping mall platform. The Naver platform collects user reviews written in various other shopping malls and displays all of them. Therefore, it was judged that the representation of review posts would be high, so it was selected as a target platform.

**Figure 5:**
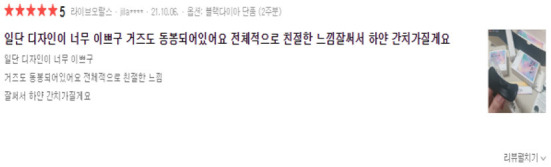
Example of a comment in Naver shopping mall platform.

**Figure 6:**
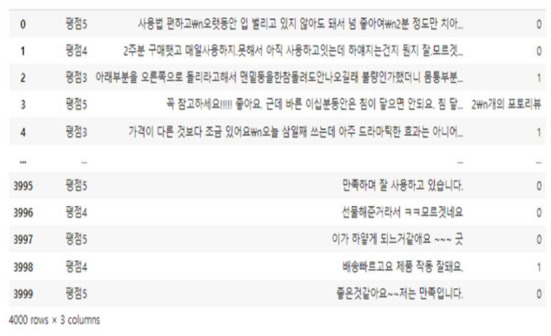
Comment dataset collected with selenium.

**Figure 7:**
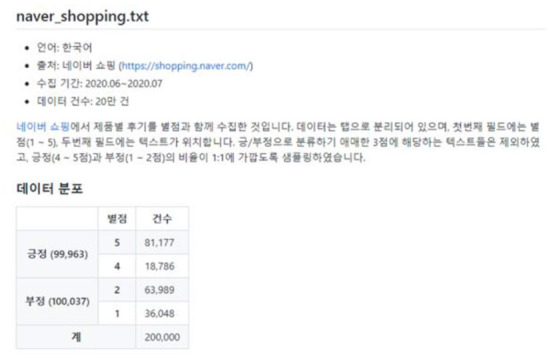
Naver shopping mall comment dataset.

Among the products in Naver Shopping, three “teeth whitening” products with more than 1,000 reviews were selected and a total of 4,000 reviews were crawled. During the preliminary survey, it was found that there were a considerable number of recruiting advertisements for jobs posting part-time reviews for teeth whitening products, so teeth whitening products were selected. Through crawling, information on the platform regarding text reviews, review ratings, numbers of image attachments, dates, and the sources of platforms could be obtained. Figures 5, 6 and are the data used for the study. Figure 7 shows basic information about the data.

### 4.2. Data preprocessing

Based on previous studies, a useful standard for determining temporary views was set. We used review ratings, numbers of attached images, positive and negative text review content, review lengths, and repetition of affirmative words as measures for additive view filtering. Natural language processing techniques were used to find out whether the review content was positive or negative and the degree of repetition of positive words (Yang and Fan, 2014).

It was confirmed that there were many datasets that required orthography correction before extracting characteristics from the review article, and to this end, special symbols and emoticons in the review were first removed. We also used the Hanspell library to correct overall spelling. In this process, in order to prevent proper nouns, such as product names included in the review from changing, this was designated as an exception and then the orthography was corrected.

During the Exploratory Data Analysis (EDA), it was confirmed that a high rating of a review did not immediately lead to a high positivity of the review, and accordingly, despite the high rating, the content of the review itself was found to be neutral or negative. To supplement this, an emotional analysis of the review data was conducted using the LSTM NN.

**Figure 8:**
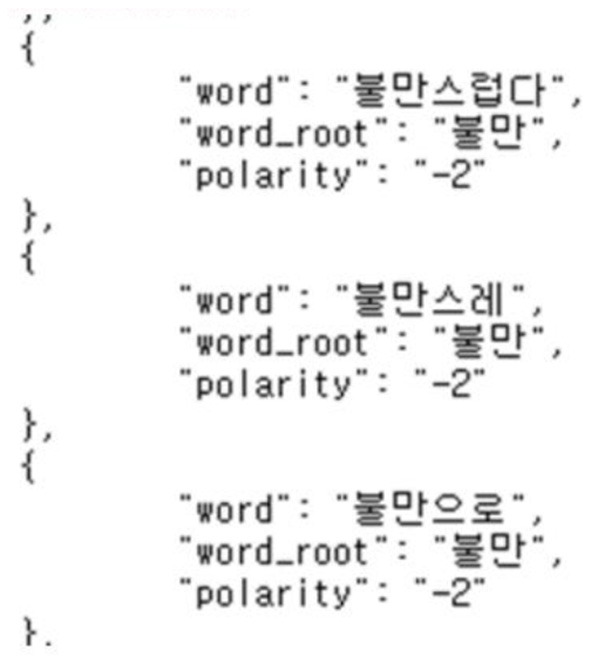
Korean emotional dictionary.

The Tokenizing process of the emotion analysis data was all performed using KoNLPy's Okt. Since Okt has the function of automatically correcting typos, it was judged that it would have a great effect on improving accuracy when applied to review data that would have many typos. Using 200,000 Naver shopping reviews from GitHub (bab2min), a model for this emotional analysis was created to add characteristics of positivity and negativity (Figure 8).

To find out the degree of repetition of positivity included in the review, the KNU Korean Emotional Dictionary in GitHub (park1200656) was used. KNU Korean Language Emotional Dictionary is a vocabulary dictionary created by the Department of Software Convergence Engineering at Gunsan University, with positive and negative scores for each word. Using the corresponding JSON file, it was identified whether there was a review token in advance, and if there was, the scores were summed to add the dictionary_score characteristics of each review.

## 5. Experiments and results

### 5.1. Unsupervised learning

**Figure 9:**
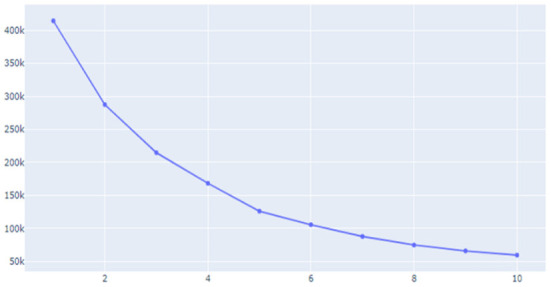
Inertial value change according to the *K*-value (1).

**Figure 10:**
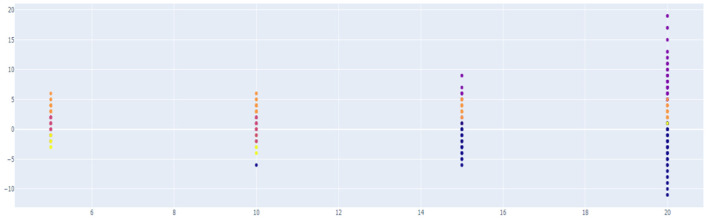
Result of hierarchical clustering.

**Figure 11:**
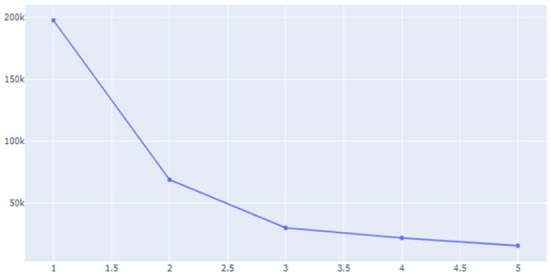
Inertial value change according to the *K*-value (2).

**Figure 12:**
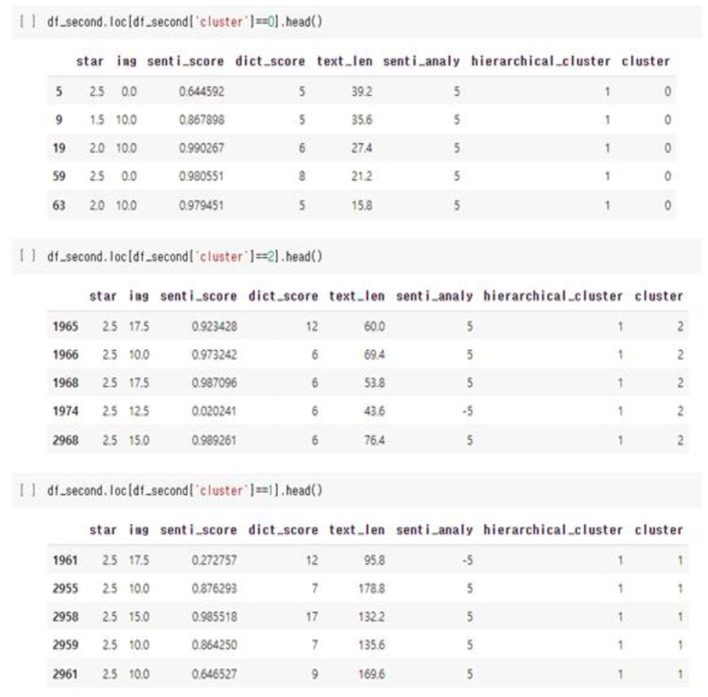
Comments in different groups of cluster.

Unsupervised learning was conducted using pre-processed data. The data used in this study were preferentially analyzed because there was no label for whether or not a temporary view was present. Since classifying the review into only two clusters, a fake review and a real review, the Inertia value is significantly higher and the classification performance is significantly lower, K-means was used to identify the appropriate number of clusters to confirm the change in the Inertia value according to the *K*-value. Through Figures 9–11, we could visualize how the dataset were clustered through out the unsupervised learning methods. The below Figure 12 shows the final result examples of unsupervised learning.

Through this, it was judged that classifying into about five clusters could lead to meaningful results, and as a result of changing the scale of each characteristic using various algorithms of cluster analysis, it was confirmed that when using Agglomerative Clustering, that is, this Hierarchical Clustering technique showed the most meaningful results. The sentence length of the classified data is the *X*-axis and the repetition degree of positive words is *Y*-axis, and the result of reducing the dimension is as follows.

Initially, previous studies assumed that the higher the sentence length and repetition of affirmative words, the higher the probability of fake reviews, so it was judged that cluster data No. 1, in purple above, was more likely to be false views. However, since the amount of cluster data is still large and extensive, clustering was conducted once again using the data.

As for group 1 above, as above, as a result of examining the trend of the change in Inertia values according to the *K*-value, it was predicted that it would be significant when classified into three clusters. As a result of classifying the data into three clusters through Hierarchical Clustering once again, it was confirmed that the “length of the review” played a large role as a classification criterion. Since it was found that the review length was not required to be excessively long during the previous part-time survey of reviews, this unsupervised learning led to the conclusion that the data of Group 2, which corresponds to the intermediate review length cluster, would be the closest to a fake review among the three groups.

However, this unsupervised learning had many limitations. It was still difficult to determine which reviews were reliably fake, and the ambiguity of the distinguishing characteristics for each group when divided into clusters also lowered the reliability of filtering.

### 5.2. Supervised learning

Next, supervised learning was conducted to determine reviews useful to consumers. It was necessary to display useful data by hand in order to conduct supervised learning with an unlabeled dataset. Accordingly, the total data was divided into 800 training sets and 3,200 test sets, and the data of the training set was manually labeled. As the length of the review was long in the process, it was judged that reviews that properly mixed the strengths and weaknesses of the product were useful data, and reviews with images or videos were also classified into more useful reviews.

After completing the above process, various algorithms were used to check the accuracy of each algorithm.

Through the above results, it was confirmed that the accuracy was high in the order of SVC, LGBM, Logical Regression, and KNN. Although there was an attempt to improve the accuracy using the Grid Search technique, it was confirmed that the 85.125% accuracy of SVC was still the highest accuracy value. It was found that the results of using the Hard Voting and Soft Voting techniques using the four models with high accuracy also remained below 85%.

The ROC Curve for the above four techniques is expressed as follows.

The AUC Score values for the above four techniques are as follows.

From the above results, it was confirmed that the AUC Score value was the highest using LGBM, followed by the SVC and KNN techniques.

Finally, we classified the data of the Test set using the SVM model, which shows high performance overall and has a lower overfitting risk compared to LGBM.

Through this, it was confirmed that reviews that provide information along with images were classified as relatively useful comments compared to text reviews.

**Figure 13:**
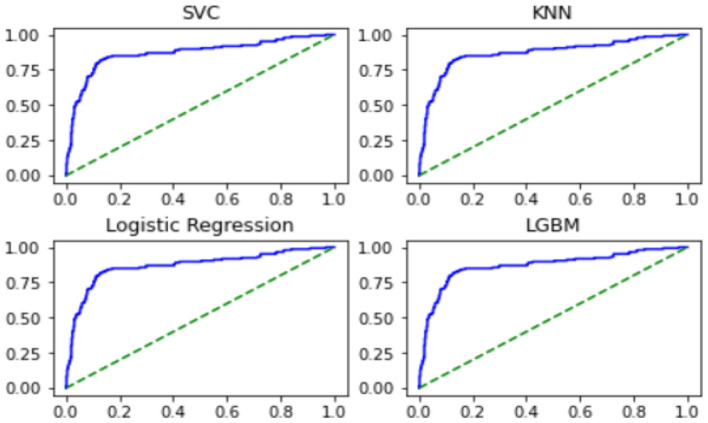
ROC curve of TOP-4 models.

**Figure 14:**
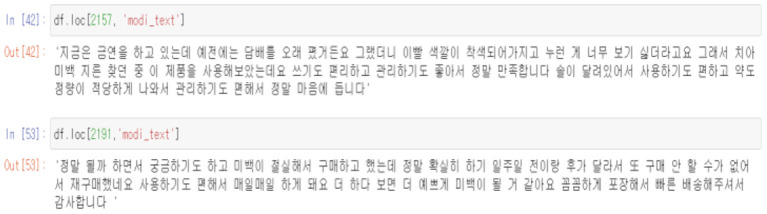
Example of reviews classified “useful”.

In addition, it could be seen that comments that do not contain information about the product because the content of the review is very short, as above, or comments that are suspected of being fake reviews because they are too long or high in positivity are also classified as non-useful comments. The below Figures 13, 14 shows both examples of Usefull and Unusefull reviews classified through our model.

## 6. Conclusion

Prior to the study, a survey found that fake reviews were abused to reduce the benefits of the entire platform participants, and accordingly, the importance of proposing fake review filtering models was recognized. In this study, through in-depth investigations such as interviews, it was confirmed that fake review marketing was actually used in the “teeth whitening” product line. Accordingly, a machine learning model was established to determine the usefulness of fake reviews and reviews through an analysis of 4,000 reviews regarding the product line.

This study used reviews of three products belonging to the “teeth whitening” product line. Among the unsupervised learning techniques, K-means, Hierarchical clustering, DBSCAN, and Spectral Cluster were used in the process of determining fake reviews among the reviews of the product. In the process of identifying useful reviews, SVC, LGBM Classifier, RandomForest Classifier, Logistic Regression, KNeighbors Classifier, Grandient Boosting Classifier, XGB Classifier, Gaussian NB, ExtraTrees Classifier, and DecisionTreeClassifier models were used for supervised learning.

As a result of the unsupervised learning of this study, there were reviews suspected of being misclassified among the reviews determined to be fake reviews, and among the reviews determined to be non-fake reviews, there were reviews suspected to be fake reviews. In addition, there was ambiguity in setting the criteria for classification. Through this, it was judged that the accuracy of fake review detection through unsupervised learning was poor.

**Figure 15:**
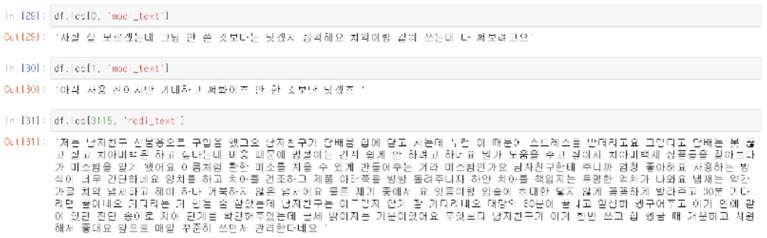
Example of reviews classified “un-useful”.

The usefulness judgment of the review showed 81–85% accuracy (Table 2) according to each analysis algorithm mentioned above as a result of supervised learning. The top four algorithms with high accuracy could probably be improved, but it is unlikely that their classification accuracies could significantly be improved in their current form. Still 3 out of the top four algorithms showed AUC score over 70% (Table 3, Figure 15).

**Table 2:** Accuracy of each algorithms used.

**Model name**	**Accuracy**
SVC	85.125
LGBM	83.874999
Random forest	83.75
Logistic regression	83.625
KNN	83.5
Gradient boosting	83.249
XGB	82.25
Gaussian NB	82.1249
Extra trees	81.875
Decision tree	81.1249

**Table 3:** AUC score of TOP-4 models.

**Model name**	**AUC score**
SVC	0.7478
KNN	0.7370
LGBM	0.7568
Logistic regression	0.6065

There are two major limitations of this study. First, BERT, which was judged to be the most suitable as an aspect of natural language processing deep learning techniques, was not able to use the model because it was a very large model with 12 GB of GPU memory recommended for learning. The second limitation concerns the data set. The crawled data was provided by Naver Shopping Mall, which seems to have already been classified once, but as a result of asking Naver whether it was screened, details of backend filtering and classification algorithms could not be identified due to in-house confidentiality. In addition, we tried to increase the accuracy by analyzing more data, but there were limitations in analyzing more data because this analysis already required significant memory resources.

In the future, it is expected that, based on this study, the accuracy of algorithms that determine the usefulness of fake reviews and reviews will be strengthened, and a model that displays real reviews near the top of the review results will be provided to the e-commerce platform. This study has shown the possibility to detect fake reviews only with Machine Learning algorithms (not Deep Learning algorithms) with precision. This will help e-commerce platforms increase service reliability, prevent information overload, prevent participants from leaving the platform, and provide better online shopping experiences to attract more consumers.

## Data availability statement

The raw data supporting the conclusions of this article will be made available by the authors, without undue reservation.

## Author contributions

All authors listed have made a substantial, direct, and intellectual contribution to the work and approved it for publication.
